# PReS-FINAL-2315: Severe cutaneous vasculitis in two patients with juvenile idiopathic arthritis and biologic therapy

**DOI:** 10.1186/1546-0096-11-S2-P305

**Published:** 2013-12-05

**Authors:** F Falcini, G Lepri, G Carnesecchi, F Bertini, M Matucci Cerinic

**Affiliations:** 1 Internal Medicine, Rheumatology Section, Transition Clinic, Florence, Italy; 2 Internal Medicine, Rheumatology Section, University of Florence, Florence, Italy

## Introduction

The management of chronic inflammatory autoimmune diseases has been revolutioned by the use of novel biologic agents achieving excellent results in the treatment of Juvenile Idiopathic Arthritis (JIA). These drugs are generally well tolerated but as they are increasingly used, the incidence of adverse reaction is more common. The most frequent are localised at the injection's site; others include: pustular folliculitis, psoriasis, hydradenitis, Sweet's syndrome, lupus-like reactions, and palm plantar pustolosis. Cutaneous vasculitis related to TNF-alpha antagonists, such as hypersensitivity and leucocytoclastic vasculitis, has been described as few case reports. Conversely, there are no reports of cutaneous vasculitis induced from other biologic agents. We report two cases of severe cutaneous vasculitis in adolescents with JIA and uveitis under biologic therapy.

## Objectives

To report cutaneous complications of biologic agents.

## Methods

From Database of all JIA patients cared at our clinic with biologic therapy we look for those with side effects and we found two patients with cutaneous vasculitis.

## Results

**Patient 1**: Male with JIA ERA onset HLA B27+ and severe bilateral uveitis since the age of 9 yrs. After NSAIDs, steroids and MTX, biologics were introduced due to persistent articular and ocular activity. The patient developed cutaneous folliculitis with fungal super infection both during Infliximab infusion, discontinued after 5 years for allergic reaction, and Abatacept stopped for inefficacy. Therefore Adalimumab was started with a good control of disease's activity, but 3 years later the patient developed skin reactions at the lower limbs. Biopsy revealed leucocytoclastic vasculitis. The drug was withdrawn and steroid pulses administered. Adalimumab was resumed after the disappearance of the lesions without further complications. **Case 2: **Girl with extended oligoarticular IJA and bilateral uveitis since the age of 2 years. After NSAIDs, steroids, MTX, and Infliximab, Adalimumab was introduced followed by inguinal and axillary skin lesions like psoriasis. Due to relapse of arthritis Abatacept was substituted to Adalimumab. At the second infusion, patient developed cutaneous reaction at the lower limbs classified by biopsy as leucocytoclastic vasculitis. The lesions improved after drug withdrawn and steroid administration.

## Conclusion

Biologics are effective and safe in JIA. As more and more widely used the adverse effects are more and more frequent. Our cases confirm the just reported vasculitic reactions under antiTNF-alpha drugs, and highlights that this event can also occur also with non-anti TNF-alpha biologic drugs.

## Disclosure of interest

None declared.

